# The Role of Social Norms in the Portion Size Effect: Reducing Normative Relevance Reduces the Effect of Portion Size on Consumption Decisions

**DOI:** 10.3389/fpsyg.2016.00756

**Published:** 2016-05-31

**Authors:** Iris Versluis, Esther K. Papies

**Affiliations:** ^1^Department of Econometrics, Erasmus School of Economics, RotterdamNetherlands; ^2^Institute of Neuroscience and Psychology, University of GlasgowGlasgow, UK; ^3^Department of Social and Organizational Psychology, Utrecht UniversityUtrecht, Netherlands

**Keywords:** portion size effect, amount served, normative influence, social norm, overweight, consumption quantity decisions

## Abstract

People typically eat more from large portions of food than from small portions. An explanation that has often been given for this so-called portion size effect is that the portion size acts as a social norm and as such communicates how much is appropriate to eat. In this paper, we tested this explanation by examining whether manipulating the relevance of the portion size as a social norm changes the portion size effect, as assessed by prospective consumption decisions. We conducted one pilot experiment and one full experiment in which participants respectively indicated how much they would eat or serve themselves from a given amount of different foods. In the pilot (*N* = 63), we manipulated normative relevance by allegedly basing the portion size on the behavior of either students of the own university (in-group) or of another university (out-group). In the main experiment (*N* = 321), we told participants that either a minority or majority of people similar to them approved of the portion size. Results show that in both experiments, participants expected to serve themselves and to eat more from larger than from smaller portions. As expected, however, the portion size effect was less pronounced when the reference portions were allegedly based on the behavior of an out-group (pilot) or approved only by a minority (main experiment). These findings suggest that the portion size indeed provides normative information, because participants were less influenced by it if it communicated the behaviors or values of a less relevant social group. In addition, in the main experiment, the relation between portion size and the expected amount served was partially mediated by the amount that was considered appropriate, suggesting that concerns about eating an appropriate amount indeed play a role in the portion size effect. However, since the portion size effect was weakened but not eliminated by the normative relevance manipulations and since mediation was only partial, other mechanisms may also play a role.

## Introduction

The size of the portion one is served has a strong and well recognized influence on the amount of food that people consume, such that more is consumed when the portion size increases (see for example Zlatevska et al., 2014; Hollands et al., 2015 for reviews). This so-called portion size effect occurs for different types of food (Rolls et al., 2004; Stroebele et al., 2009; Burger et al., 2011), in different settings (Diliberti et al., 2004; Wansink and Kim, 2005) and for different people (Wansink and van Ittersum, 2007). Less is known, however, about why the portion size effect occurs. Although there are various reviews of the portion size effect and its possible causes (see for example: Ledikwe et al., 2005; Steenhuis and Vermeer, 2009; Livingstone and Pourshahidi, 2014; Benton, 2015; English et al., 2015; Herman et al., 2015), these reviews all indicate that no definitive conclusions can be drawn and that more research is needed on the mechanisms of the portion size effect.

Two recurring explanations for the portion size effect are visual cues (Benton, 2015; English et al., 2015; Herman et al., 2015) and appropriateness (Rolls et al., 2002; Herman and Polivy, 2005, 2008; Wansink and van Ittersum, 2007; Steenhuis and Vermeer, 2009; Herman et al., 2015). With regard to the first explanation, consumption and satiety judgments are believed to be influenced by what we see. As our size judgment is prone to various biases (Chandon and Wansink, 2007; Geier and Rozin, 2009; Ordabayeva and Chandon, 2013), this can lead to overconsumption from large portions. However, much is still unclear about how exactly visual cues impact consumption quantity decisions (Benton, 2015; English et al., 2015; Herman et al., 2015) and more research is needed before this explanation can be formally tested. We therefore focus on the explanation that has been most dominant in the literature: appropriateness (Herman et al., 2015).

According to the appropriateness explanation the amount of food provided serves as a cue for what is an ‘appropriate’ amount to eat (Rolls et al., 2002; Herman and Polivy, 2005, 2008; Wansink and van Ittersum, 2007). More specifically, according to Herman et al. (2003) and Herman and Polivy (2014), portion sizes act as upper limits for intake and define how much can be maximally eaten without being perceived as an excessive eater. Although we often eat for hedonic enjoyment, excessive eating behavior can be associated with several negative stereotypes which people want to avoid, such as having low self-control (Puhl and Brownell, 2001) or as being less attractive (Chaiken and Pliner, 1987; Bock and Kanarek, 1995). Eating the amount of food that is provided may be seen as acceptable consumption behavior, especially when the amount is determined by someone who can be expected to have knowledge about the “correct” portion size, such as the chef in a restaurant, the manufacturer of prepared meals, or the researcher in a lab. The portion size may thus provide indirect information about what is socially acceptable eating behavior. As the portion increases, so does the amount that people maximally allow themselves to eat, resulting in the portion size effect.

In the current paper, we argue that if the portion size indeed acts as a social norm and as such communicates how much is maximally appropriate to eat, then the effect should weaken when people do not actually believe that the portion size communicates a norm that is relevant to them. We tested this hypothesis by leading participants to believe that the portion sizes they were presented with were either based on the values and behavior of a relevant social group or not, and examined the effect of this manipulation on the effect that the portion sizes had on participants’ eating intentions.

### The Influence of Social Norms on Eating Behavior

People are influenced both by what other people do and by what other people think (Deutsch and Gerard, 1955). Cialdini et al. (1991) have distinguished between injunctive norms, which can be described as the perception of what behaviors others approve or disapprove of, and descriptive norms, which can be described as the perception of what most people actually do. Injunctive norms have been shown to influence both attitudes (for example political attitudes, see Smith and Louis, 2008) and behavior (for example littering, see Cialdini et al., 1990; Reno et al., 1993). In the area of eating behavior, the impact of descriptive norms has been studied extensively (for reviews, see Cruwys et al., 2015; Higgs, 2015; Vartanian et al., 2015) and it has been shown that the amount consumed by a person is heavily influenced by the amount others consume (Conger et al., 1980; Leone et al., 2007; Hermans et al., 2012; Florack et al., 2013). Vartanian et al. (2013) for example showed that food intake was strongly influenced by how much others ate or were believed to have eaten. Furthermore, this effect of others’ consumption on intake was mediated by the amount that was perceived to be appropriate to eat. These influences even occur when the other person is not physically present or when a so-called remote confederate design is used. In the latter, the information about someone else’s consumption is provided for example in the form of written information or through other, more subtle cues, such as by leaving candy wrappers from the “previous participant” on the table (Pliner and Mann, 2004; Feeney et al., 2011; Robinson et al., 2013; for a review, see: Robinson et al., 2014). In addition, research has shown that both injunctive and descriptive norms are associated with healthy eating behavior, even if this is typically assessed through correlations among self-report measures (e.g., Armitage and Conner, 1999; Povey et al., 2000; Rivis and Sheeran, 2003). In sum, normative cues suggesting appropriate behavior heavily influence people’s eating behavior, even when no other people are physically present.

At the same time, people are not equally influenced by everyone. According to self-categorization theory, social influence is dependent on the social identity of the source and target of influence (Tajfel, 1978; Turner et al., 1987; Turner and Oakes, 1989; Turner, 1991). Specifically, people categorize themselves as belonging to certain in-groups, which, depending on the situation, can be very broad, e.g., women, or very narrow, e.g., chess club members (Oakes, 1987). In-group members are viewed as being similar to the self on relevant, salient dimensions, and hence their actions and opinions are viewed as relevant and important (Turner, 1991; Haslam and Turner, 1992). Therefore, when a specific in-group identity is made salient, people will be influenced by the actions and expectations of members of this in-group (Platow et al., 2005). Those who do not belong to the in-group, the so-called out-group members, are considered less relevant to the own identity, and hence their actions and expectations affect people to a lesser degree (Turner, 1991; Haslam and Turner, 1992).

The moderating effect of the source of a social norm has clearly been demonstrated in the domain of consumption behavior. Cruwys et al. (2012) for example showed that students only modeled popcorn consumption of students from their own university and not of students from another university. Similarly, Stok et al. (2012) told participants that either the minority (27%) or the majority (73%) of Dutch students ate “sufficient” fruit. They found that considerably more students intended to eat sufficient fruit themselves when they received majority norm information than when they received minority norm information. Hermans et al. (2008) and McFerran et al. (2010) showed in a naturalistic setting that people only modeled the consumption and food choice behavior of others when they had a body type that was similar to their own. Finally, Oyserman et al. (2007) and Berger and Heath (2008) showed that when the social norm, such as healthy or unhealthy eating, was communicated by out-group members, this even led to reactance against the norm, leading in-group members to adopt norm-incongruent eating behaviors.

Here, we suggest that if the portion size provides normative information about the appropriate amount to eat, its effect on consumption decisions should be reduced when people learn that the portion size is based on the opinion or behavior of others who are considered relatively less relevant to oneself.

### Current Research

In the current research we manipulated the normative relevance of the portion size by telling participants that it was based on the behavior of an in-group or an out-group (pilot experiment; based on Cruwys et al., 2012), and by providing information that it was approved of by a minority or majority of people similar to the participants (the main experiment; based on Smith and Louis, 2008; Stok et al., 2012).

The paradigm used in the current studies was based on Marchiori et al. (2014) who first asked participants to imagine a certain portion of food that they were served in a certain situation, and to indicate if they would eat more or less than the specified portion. In our study, we added the information that this portion size was based on the behavior of a certain social group or approved by a certain social group. Then, participants were asked how much exactly they expected to consume in the pilot experiment and expected to serve themselves in the main experiment. These estimations served as the dependent variables. Importantly, in previous research, these intake estimations were found to be affected by the initial portion size that participants had been asked to imagine (Marchiori et al., 2014). Here, we tested whether this effect could be modulated by manipulating the normative relevance of the initial portion size. Based on recent studies that show that the portion size effect might already occur even before eating commences, hence, when people decide how much to serve themselves or how much they are going to eat (Marchiori et al., 2014; Robinson et al., 2015), we decided to focus our research on these consumption decisions. Indeed, various studies have shown that the consumption estimations made using food pictures (Fay et al., 2011; Wilkinson et al., 2012; Brunstrom, 2014) or fake foods (Bucher et al., 2012) closely relate to what people actually eat. Hence, we examined how manipulating the normative relevance of the portion size impacted participants’ consumption decisions.

## Pilot Experiment

### Method

#### Design and Participants

The experiment had a 2 (normative relevance: in-group vs. out-group; between participants) × 2 (portion size: small vs. large; within participants) mixed design. Participants were provided with a portion size that allegedly was based on the eating behavior of students from their own university (in-group, Erasmus School of Economics), or students of literature from another university (out-group, University of Amsterdam).

The sample consisted of 63 economics students of a Dutch university who followed an advanced market research class and were asked during a lecture whether they would like to voluntarily fill in a short questionnaire.

#### Procedure

The experiment was administered as a paper-and-pencil questionnaire during a lecture. We told participants that the purpose of the questions was to determine how we could best phrase questions about consumption (Marchiori et al., 2014). Participants indicated how much they expected to consume of two dinner foods (pasta, soup) and two snack foods (mini chocolate chip cookies, cheese cubes). Expected consumption was indicated in grams for the pasta, in milliliters for the soup, and in pieces for the cookies and cheese cubes. For soup, participants were told: “Imagine that you are going to eat soup tonight. Will your consumption be higher or lower than [200/600] milliliter of soup? The quantity of [200/600] ml is based on research among [students from Erasmus School of Economics/literature students from University of Amsterdam]. [200/600] ml is the amount that these students on average served themselves.” Participants indicated if they would eat more or less than the specified amount and then answered the question: “How much soup will you consume? Please provide your answer in milliliters.” Each participant was presented with a large and small portion for the dinner foods and a small and large portion for the snack foods. The participants either saw a large portion for the pasta and cookies and a small portion for the soup and cheese cubes or vice versa. The order in which the foods were presented was counterbalanced with the constraint that the participants always saw the dinner food questions right after each other and the snack food questions right after each other.

We based the small and large portion for each of the foods on the average amount consumed per consumption occasion in the Netherlands (Dutch National Food Consumption Survey, 2007–2010). As displayed in **Table 1**, depending on the food, the small portion was 20–40% smaller than the average consumption amount. The large portion was about three times as large as the small portion.

**Table 1:** Foods and portion sizes in the pilot experiment.

	Occasion	Average consumption quantity^a^	Small portion	Large portion
Pasta	Dinner	170 g	120 g	400 g
Soup	Dinner	260 ml	200 ml	600 ml
Mini chocolate cookies	Snack	5 cookies	3 cookies	10 cookies
Cheese cubes	Snack	3 cubes	2 cubes	7 cubes

*^a^Based on the Dutch National Food Consumption Survey, 2007–2010.*

Participants then completed a number of other measures, including the extent to which they identified with the in-group or out-group using the following two statements measured on a 7-point scale: “I identify with [in-group/out-group]” (Stok et al., 2012) and “I feel a connection to [in-group/out-group],” α = 0.81 for the in-group, and α = 0.79 for the out-group. Finally, the researcher then collected the questionnaires and participants were debriefed.

### Results

#### Manipulation Check

As expected, participants identified themselves more strongly with economics students from Erasmus School of Economics (*M* = 4.7, *SD* = 1.3) than with literature students from University of Amsterdam (*M* = 2.5, *SD* = 1.3), *t*(61) = 6.92, *p* < 0.01. Furthermore, across the four foods, on average 72% of participants indicated they would eat more than the provided portion when this portion was small, indicating that this portion was indeed perceived as being small. Similarly, 69% indicated that they would eat less than the provided portion when this portion was large. This suggests that both our manipulations were successful.

#### Portion Size Effect

We calculated the portion size effect for each participant by first standardizing all consumption amounts, and then subtracting the standardized consumption of the foods shown with a small portion size from the standardized consumption of the foods shown with a large portion size. We used a *t*-test to compare the magnitude of the portion size effect in the in-group and out-group condition. The average expected consumption of the four foods can be found in **Table 2**. Confirming our main hypothesis, the portion size effect was indeed significantly greater in the in-group condition than in the out-group condition, *t*(50) = 2.29, *p* = 0.03, $\eta_{p}^{2}$ = 0.09.

**Table 2:** Average expected consumption of each food across experimental conditions and the magnitude of the portion size effect in grams for pasta, in milliliter for soup, and in pieces for cookies and cheese cubes.

	In-group condition			Out-group condition		
	Small portion	Large portion	Portion size effect	Small portion	Large portion	Portion size effect
Pasta	176.3 (66.9)	358.7 (164.7)	182.4 (260 kcal)	166.9 (102.1)	300.3 (112.8)	133.4 (190 kcal)
Soup	285.7 (130.7)	584.4 (198.9)	298.7 (195 kcal)	334.4 (181.8)	421.9 (143.7)	87.5 (55 kcal)
Cookies	4.2 (2.3)	6.9 (4.7)	2.7 (45 kcal)	6.1 (5.1)	6.0 (3.3)	0
Cheese cubes	4.7 (4.1)	5.3 (2.6)	0.6 (35 kcal)	5.8 (3.4)	5.5 (2.9)	0

*Calories are estimated based on the Dutch National Food Consumption Survey, 2007–2010. Standard deviations are provided in parentheses for the average expected consumption amounts.*

### Discussion

The results of this pilot experiment suggested that the magnitude of the portion size effect indeed depended on the relevance of the portion size as a social norm. Specifically, the portion size effect, assessed as expected consumption, was weaker when the portion size had allegedly been based on the eating behavior of an out-group than when it had been based on the in-group. Hence, when the portion size was based on the eating behavior of a social group with which participants did not strongly identify, they were less inclined to use it as a reference point to determine their own expected consumption.

## Main Experiment

As results from the pilot experiment were promising, we next conducted a full experiment to further investigate the role of social norms in the portion size effect.

In this experiment, we recruited a sufficiently large sample for a full between-participants analysis of the effect of portion size and the normative relevance manipulation. Furthermore, as students are a very specific group whose behaviors and attitudes are not always representative of older adults (Sears, 1986), we now recruited a general sample of the Dutch population.

Importantly, we now manipulated the normative relevance of the portion size by providing information about whether it was allegedly approved of by a majority or minority of people similar to the participants. This type of normative claim has both descriptive and injunctive elements, as it suggests that a minority or a majority of people that are relevant to the participant approve of the portion. According to Herman et al. (2003) and Herman and Polivy (2014), people mainly use the portion size as a reference point in their consumption decision to gain social approval, relying on the portion size as an indicator of the maximum one can eat without coming across as an excessive eater. Thus, manipulating to what extent relevant others allegedly *approve* of the portion size should be a direct test of the notion that people use the portion size as an indicator of what is socially acceptable.

In the current experiment, we operationalized this by providing information about the percentage of Dutch women who allegedly considered the presented portion to be appropriate. We recruited only women, as they tend to be more concerned about their eating behavior than men (Mori et al., 1987; Divine and Lepisto, 2005). Furthermore, we asked how much participants expected to serve themselves rather than how much they would consume, as this judgment might be easier to make in an online setting.

This experiment was also designed to include mediation analysis to test whether considering a larger portion “appropriate” is an underlying mechanism for the finding that larger portions increase intake (see also Vartanian et al., 2013; Kerameas et al., 2015). To this end, we asked participants to indicate the amount that they considered appropriate to serve themselves in each consumption situation, and we included this variable in a moderated mediation analysis. Here, we expected that the effect of a large portion increasing intake would be mediated by participants finding a larger portion appropriate. At the same time, this effect should be weaker in the condition of low normative relevance where participants learned that only a small percentage of similar others approved of the portion.

Finally, we also examined the effect of adding pictures of the foods to facilitate portion size estimation. People find it difficult to interpret food amounts in grams (Faulkner et al., 2012). Since in the pilot experiment, expected consumption of two of the foods had to be estimated in grams and the portion size information was also only provided in grams, this might have made the estimation task difficult. This difficultly could have artificially strengthened the portion size effect, as participants could not rely on visual estimations of food amounts that they are more familiar with. Therefore, we predicted in the main experiment that providing a picture of the food portions would weaken the portion size effect, and that it would also strengthen the moderating effect of the normative relevance manipulation.

### Method

#### Design

The experiment had a 2 (portion size: small vs. large) × 2 (normative relevance: minority vs. majority) × 2 (picture of portion size: absent vs. present) between-participants design.

#### Participants

The sample consisted of Dutch females between 18 and 55 years old who had eaten the foods in the study at least once in the past. All participants were members of the Dutch consumer panel of panel agency GMI and provided informed consent to take part in the study. Participants were not allowed to continue with the questions if they could not remember the normative relevance manipulation correctly.^1^ We used this screening procedure to prevent individuals from participating in the study who would not carefully read our instructions and questions, which can be a problem in research with online participation (see Oppenheimer et al., 2009; Berinsky et al., 2014). A total of 347 participants completed the questionnaire. Nine participants were excluded from analyses because of low data quality, which either meant that they gave the same answer to at least 26 of the 28 agree/disagree and true/false statements or filled in the questionnaire in less than 5 min [mean completion time was 15 min (*SD* = 10)]. Furthermore, another 14 participants were excluded because they wrongly interpreted the expected amount served questions for pasta and rice. We specifically asked participants to indicate the amount including other ingredients such as vegetables, meat and sauce. Some participants, however, indicated in the open-ended questions that they provided their answers regarding the amount of (dry) pasta or rice excluding any other ingredients. Hence, these participants were excluded from analyses. Last, we excluded three outliers, as the amount they expected to serve lay more than 5 SD from the mean amount served for one or more of the foods. This led to a final sample of 321 participants, who had a mean age of 36.7 (*SD* = 10.6).

#### Foods

We included two dinner foods (pasta; Indonesian fried rice) and two snack foods (mini ginger cookies, in Dutch “kruidnoten”; potato chips). The order in which the foods were presented was randomized. The expected amount served was asked in grams for each food. For the pasta and Indonesian fried rice it was made clear that the reference amount included other ingredients such as sauce, vegetables, and meat. We based the small and large portion for each of the foods on the average amount consumed per consumption occasion in the Netherlands (Dutch National Food Consumption Survey, 2007–2010) and on the portion size information on the pack of the manufacturer. Depending on the food, the small portion was 40–50% smaller than the average consumption amount. Small portions were somewhat smaller than in the pilot experiment to make sure they would look sufficiently small on the pictures. For the dinner foods, the large portion was three times as large as the small portion; for the snack foods, it was four times as large (see **Table 3** for all amounts). For the condition which included pictures of the food portions, we photographed each food on a white plate. As a size reference, we put a pen and a glass of water next to the plate. For the dinner foods, we also included a knife and fork. For example pictures, please refer to the Supplementary Materials.

**Table 3:** Foods and portion sizes in the main experiment.

	Occasion	Average consumption amount^a^	Small portion	Large portion
Pasta	Dinner	350 g	200 gram	600 g
Fried rice	Dinner	300 g	170 g	500 g
Mini ginger cookies	Snack	25 g	15 g	60 g
Chips	Snack	40 g	20 g	80 g

*^a^Based on the Dutch National Food Consumption Survey, 2007–2010 and manufacturer information.*

#### Procedure

Participants were recruited by panel agency GMI, who also provided them with a small monetary compensation for participation. The questionnaire was administered in Dutch. Participants were randomly allocated to 1 of the eight experimental conditions. After the screening questions, participants were told that we wanted to learn more about their eating habits and food preferences. Participants then read a brief text about previous portion size research that had been conducted among Dutch women and that had shown that the portion sizes the participants were about the see were regarded as appropriate by [10%/80%] of Dutch women. After participants were asked to recall this percentage, they were presented with the scenarios in which we asked how much they would serve themselves of four different foods. For pasta, participants were told: “Imagine that you are going to eat pasta tonight. There is more than enough and you serve yourself a portion. In previous research we asked Dutch women what they think about a portion of [200/600] grams of pasta. According to this research, [only 10%/as much as 80%] of women find this portion appropriate. Would you serve yourself more or less than [200/600] grams of pasta? Please note that you should indicate the amount of pasta including sauce and other ingredients.” Participants indicated if they would serve more or less than the specified amount and then answered the question: “How much pasta would you serve yourself? Please provide your answer in grams.” In the picture condition, a picture of the portion of [200/600] grams was included. Thus, participants could freely indicate how much pasta they would eat, and this could be more or less than the portion described in the scenario. This procedure was repeated for all foods.

Participants then completed a number of other measures (see below), after which they were thanked and debriefed.

#### Other Measures

The measures that are included in the subsequent analyses are listed here. For all other measures please refer to the Supplementary Material. All scales are 7-point scales, unless stated otherwise. We first asked age and frequency of consumption of the four foods in the study. After the consumption scenarios, participants were asked to explain for two foods in an open ended question how they had determined their expected amount served. Participants then indicated how difficult or easy it had been for them to indicate their expected amount served of all four foods. In case participants had been shown a photo of each of the food portions, they indicated how attractive the foods on each photo looked. All participants then indicated liking of each of the four foods. Participants then again saw the scenarios, only now we asked them to not indicate the amount they expected to serve themselves, but the amount that they thought would be appropriate to serve in this situation, which served as the mediator in the moderated mediation analyses. Next, we asked how believable they found the cover story which contained the normative relevance manipulation. Participants then moved on to the dietary restraint subscale of the Three Factor Eating questionnaire (Stunkard and Messick, 1985; α = 0.88). Next, participants indicated if they were currently trying to lose weight (yes, a bit, no) and completed the perceived self-regulatory success scale (Fishbach et al., 2003; α = 0.79). To measure social identification with the in-group/out-group we also included items from the Social Identification Scale by Leach et al. (2008), “I feel a bond with Dutch women,” “I feel solidarity with Dutch women,” “I think that Dutch women have a lot to be proud of,” “I have a lot in common with the average Dutch woman,” and based on Stok et al. (2012) we included “I identify with Dutch women,” with α = 0.90. Robinson et al. (2011) showed that the degree of social modeling was moderated by trait self-esteem, with social modeling being higher when self-esteem was low. We therefore also included the 10-item self-esteem scale of Rosenberg (1965), α = 0.90. We assessed current hunger by two statements (‘How hungry are you at this moment’; ‘How much could you eat right now’; α = 0.85). Next, participants provided their gender, weight and height. Finally, participants wrote down what they thought the purpose of the study was, after which they were debriefed and could write down comments.

#### Randomization Check

Using an ANOVA with portion size, normative relevance and presence of a picture as factors, we found no significant differences across conditions with regard to BMI, dietary restraint, current dieting behavior, attractiveness of the food pictures, hunger, self-esteem, and liking and consumption frequency of the foods (all *ps* > 0.05). The extent to which participants identified themselves with Dutch women varied per portion size condition, *F*(1,313) = 4.49, *p* = 0.03, with identification being lower in small portion size condition (*M* = 3.99, *SD* = 1.21) than in the large portion size condition (*M* = 4.28, *SD* = 1.26). Note that the answer to this question might have been influenced by the portion size and social information condition the participant was presented with.

### Analysis

We standardized expected consumption of each food and calculated the average expected consumption across the four foods. To directly test our hypothesis that the portion size effect is smaller when the normative relevance of the portion size is high compared to when it is low (see Hancock and Klockars, 1996), we conducted a simple main effects analysis to determine whether the portion size effect was stronger in the majority condition than in the minority condition. We then performed 2 × 2 × 2 ANOVA with portion size, normative relevance and presence of a portion size picture as factors.

To examine potential effects of other variables such as BMI and self-regulatory success, we included them in a General Linear Model, and in case of a significant moderating influence, we used simple slopes analyses to further examine their effect on portion size and normative relevance. We used repeated measures ANOVA to determine if the effect of normative relevance differed across the four foods. As Mauchly’s test indicated that the assumption of sphericity was violated, χ^2^(5) = 147.16, *p* < 0.01, we used a Greenhouse–Geisser degrees of freedom correction.

Finally, we used a bootstrapping procedure (Preacher and Hayes, 2008) to perform a moderated mediation analysis, in which we analyzed whether the relation between portion size and expected amount served was mediated by the amount that was considered appropriate, and whether this mediation effect differed in the minority and majority condition.

### Results

#### Manipulation Check

The large portion was considered to be significantly larger than the small portion, all *p*s < 0.01. Furthermore, as expected, it was considered more difficult to estimate the expected amount served when no picture was included than when a picture was included, all *p*s < 0.02. Furthermore, across the four foods, on average 71% of participants indicated that they would eat more than the provided portion size when this portion size was small, indicating that this portion was indeed perceived as being small. Similarly, 72% indicated that they would eat less than the provided portion size when this portion was large.

#### Portion Size Effect

The average expected amount served of the four foods can be found in **Table 4**. Simple main effects confirmed our hypothesis that although the portion size effect was significant in both conditions, it was weaker in the minority condition *F*(1,313) = 74.63, *p* < 0.01, $\eta_{p}^{2}$ = 0.19, than in the majority condition, *F*(1,313) = 136.10, *p* < 0.01, $\eta_{p}^{2}$ = 0.30. The estimated mean difference of standardized serving scores between the small and large portion in the minority condition was *M* = 0.78 (*SE* = 0.09), and in the majority condition *M* = 1.11 (*SE* = 0.10). Further follow-up analyses showed that this was due to low normative relevance reducing how much participants would serve themselves in the large portion condition, *F*(1,313) = 9.55, *p <* 0.01, $\eta_{p}^{2}$ = 0.03. Normative relevance had no effect in the small portion condition, *F*(1,313) = 0.21, *p* = 0.65, $\eta_{p}^{2}$ < 0.01. Taken together, the portion size effect was smaller in the minority condition than in the majority condition, due to the decrease in expected amount served in the large portion condition.

**Table 4:** Average expected amount served of each food in grams, and the magnitude of the portion size effect across experimental conditions in grams and in calories.

	Majority condition (80% approves)			Minority condition (10% approves)		
	Small portion	Large portion	Portion size effect	Small portion	Large portion	Portion size effect
Pasta	213.5 (84.7)	434.0 (116.7)	220.5 (300 kcal)	235.6 (87.6)	362.9 (157.1)	127.3 (170 kcal)
Fried rice	198.5 (65.4)	387.6 (120.0)	189.1 (300 kcal)	209.0 (72.7)	348.8 (132.5)	139.8 (225 kcal)
Mini ginger cookies	39.9 (32.5)	73.1 (45.4)	33.2 (145 kcal)	40.3 (35.2)	64.4 (43.1)	24.1 (105 kcal)
Chips	55.1 (50.5)	86.4 (44.6)	31.3 (170 kcal)	52.0 (33.4)	80.9 (46.1)	28.9 (160 kcal)

*Calories are estimated based on the Dutch National Food Consumption Survey, 2007–2010 and manufacturer information. Standard deviations are provided in parentheses for the average expected consumption amounts.*

**FIGURE 1:**
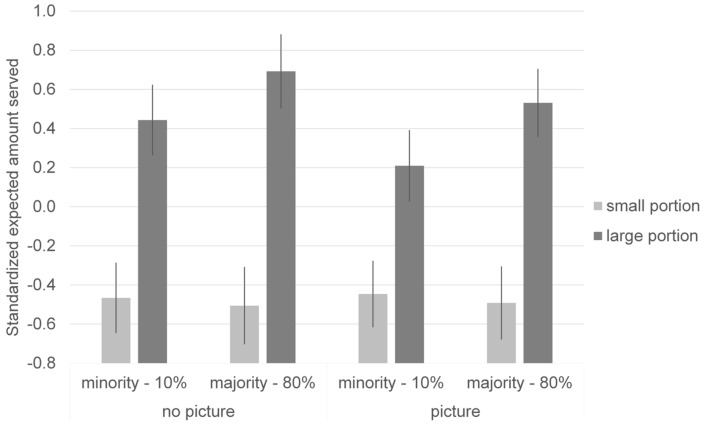
**Standardized average expected amount served across the portion size, presence of a picture and normative relevance conditions**.

This was confirmed by the omnibus ANOVA including portion size, normative relevance and presence of a picture, which revealed a main effect of portion size, *F*(1,313) = 207.54, *p* < 0.01, $\eta_{p}^{2}$ = 0.40 and a significant interaction between portion size and normative relevance, *F*(1,313) = 6.23, *p* = 0.01, $\eta_{p}^{2}$ = 0.02. This effect is displayed in **Figure 1**. In addition, normative relevance had a marginally significant main effect on expected serving size, *F*(1,313) = 3.41, *p* = 0.07, $\eta_{p}^{2}$ = 0.01, with expected amount served being generally higher in the majority condition than in the minority condition.

Contrary to our predictions, the presence of a picture of the portion size had no main or interaction effects, all *p*s > 0.10.

#### Additional Analyses

Dietary restraint, perceived self-regulatory success, BMI, hunger, social identification with Dutch women, and self-esteem did not moderate the effect of portion size, normative relevance nor the interaction between normative relevance and portion size (all *p*s > 0.10).

Believability of the cover story was moderate, with *M* = 3.8 (*SD* = 1.47) measured on a 7-point scale. There was an interaction between believability of the cover story and portion size, *F*(1,309) = 11.20, *p* < 0.01, $\eta_{p}^{2}$ = 0.03, such that the portion size effect was considerably stronger when believability was high (1 SD above the mean) than when believability was low (1 SD below the mean) (Aiken and West, 1991).

The repeated measures ANOVA showed that the specific food item did not moderate the main effect of normative relevance, *F*(2.42,757.18) = 0.17, *p* = 0.88, $\eta_{p}^{2}$ < 0.01, and neither did it moderate the interaction between portion size and normative relevance, *F*(2.42,757.18) = 2.31, *p* = 0.09, $\eta_{p}^{2}$ = 0.01.

#### Mediating Effect of Appropriate Consumption

**FIGURE 2:**
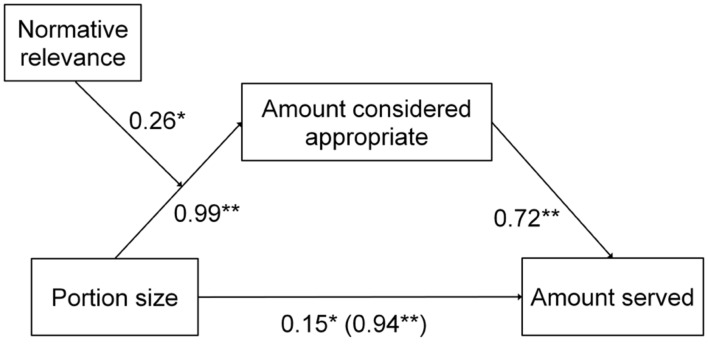
**Unstandardized regression coefficients of the moderated mediation analysis.** The coefficients in parentheses denote the coefficient for the direct effect of portion size on amount served. *p < 0.05 **p < 0.01.

The moderated mediation analysis confirmed that in both the minority and majority condition, the amount that was considered appropriate mediated the relation between portion size and expected amount served, with the indirect effect respectively being *B* = 0.89 (*SE* = 0.08), 95% CI [0.75, 1.07] in the majority condition, and *B* = 0.71 (*SE* = 0.08), 95% CI [0.57, 0.88] in the minority condition. A significant moderation effect showed that in line with our hypothesis, the indirect effect was stronger in the majority condition than in the minority condition, *B* = 0.19 (*SE* = 0.09), 95% CI [0.02, 0.35]. As can be seen in **Figure 2**, the stronger indirect effect in the majority condition can be attributed to the moderating effect of social information on appropriate intake, with the effect of portion size on the appropriate amount being stronger in the majority than in the minority condition. The direct effect of portion size on expected amount served was also significant, *B* = 0.15 (*SE* = 0.07), 95% CI [0.02, 0.29].

In sum, the amount that was considered appropriate partially mediated the relation between portion size and the expected amount served. Furthermore, the influence of portion size on the amount that was considered appropriate, and therefore on the amount served, was stronger when a majority found the portion appropriate than when a minority found it appropriate. In other words, a larger portion led to larger expected servings because larger servings seemed appropriate, but especially when a majority approved of the large portion.

### Discussion

The results of the main experiment provided further evidence that the magnitude of the portion size effect depends on the relevance of the portion size as a social norm. The portion size effect was weaker when the portion size was allegedly approved of only by a minority (10% of women) than when it was approved of by a majority (80% of women). Hence, when the normative relevance of the portion size was reduced, participants relied less on it to determine how much to eat.

The portion size effect was reduced in the minority condition mainly through a *reduction* in expected amount served in the large portion condition, and not from an *increase* in the amount served in the small portion condition. In other words, participants expected to serve themselves less when a large portion was described in the scenario and only a minority approved of this large portion, but did not expect to serve themselves more when a small portion was described in the scenario and when only a minority approved of this small portion. Again, it is important to realize that participants could indicate freely how much they would eat, and their consumption decision was not constrained by a specific food portion physically present, as in portion size studies measuring actual eating behavior. Our finding that the effect of normative relevance occurred only with regard to large portions is in line with Herman et al. (2003) who suggest that people are mainly concerned about eating too much, rather than too little. This concern might be even greater for women, as positive stereotypes are attached to women who eat small amounts, such as being attractive and feminine (Vartanian et al., 2007).

We should note that effect size of the normative relevance manipulation was rather small. In addition, even though the portion size effect was smaller in the minority condition than in the majority condition, it remained significant and substantial. Hence, even when the portion size was not considered as very informative, participants still used it to determine the amount they expected to serve themselves. Hence, other mechanisms in addition to social appropriateness might also play a role in the portion size effect.

The moderated mediation analysis further confirmed our hypothesis that participants used the portion size to determine how much was appropriate to serve themselves, and chose an amount similar to what they considered appropriate. Furthermore, as we expected, participants were more inclined to base the amount they found appropriate on the portion size when the majority approved of the portion size than when it was approved of by only a minority. This finding thus provides further support for the claim that the portion size provides normative information, indicating how much is appropriate to eat (Rolls et al., 2002; Herman and Polivy, 2005, 2008; Wansink and van Ittersum, 2007). At the same time, the mediation effect was only partial and people thus seem to have other reasons as well to use the portion size as a reference point to determine consumption, besides the desire to eat appropriately.

We should note that the mediating effect might have been somewhat inflated due to the way we measured the amount that was considered appropriate. The question format was the same as the format with which we measured expected amount served. The answers given to the expected amount served questions might have been highly accessible while participants were answering the amount appropriate questions, leading to answers that were very similar. Hence, other reasons for people to base their consumption on the portion size besides eating appropriately, could play an even bigger role than suggested by the current results.

A potential limitation of this experiment is the sensitivity of this type of research to demand effects. To prevent such effects as much as possible, we selected an online consumer panel that usually completes marketing studies for companies, rather than for universities. These participants were thus unfamiliar with experimental research in general, and with research focusing on eating behavior in particular. Indeed, when asked to write down the purpose of the study, only 3 out of 321 participants correctly guessed the purpose, making it unlikely that our results were driven by demand effects.

We had expected that the portion size effect would be stronger when the portion size information was only provided in grams without any visual information, but found no evidence to support this. The portion size effect was equally strong when a picture of the portion was shown than when no picture was shown. We did find a stronger portion size effect among participants who found the cover story more convincing, which may again confirm that the portion size effect was dependent on whether the portion size information was considered relevant for the consumption decision. Alternatively, it is also possible that participants who did not really believe the cover story might have felt they were being tricked by the researcher and as a result provided less reliable answers. Further research should continue to develop strong and convincing manipulations of the social relevance of presented food portions.

## General Discussion

One experiment and a small pilot experiment examined the role social norms play in the portion size effect. Building on previous suggestions that the portion size signals how much is “appropriate” to eat (Rolls et al., 2002; Herman and Polivy, 2005, 2008; Wansink and van Ittersum, 2007), we hypothesized that the magnitude of the portion size effect would depend on whether a given portion is perceived to be relevant as a social norm. We presented participants with a small or large portion of different foods and asked them to indicate how much they expected to eat or to serve themselves. As expected, the presented portion size strongly influenced the expected amount consumed and served, but this effect was moderated by the normative relevance of the portion size. The portion size effect was weaker when the portion size was allegedly based on the behavior of an out-group or when it was approved of by only a minority. Our findings further suggested that larger portions increased expected servings because larger servings seemed appropriate, but again this was especially the case if a majority approved of the larger portion.

Our research contributes to the literature on social norms and its effects on eating behavior. More specifically, in line with Cruwys et al. (2012) and Stok et al. (2012) we show that the source of a social norm has a considerable impact on the extent to which this social norm is taken into account when making consumption decisions. We also conceptually replicated earlier findings from Vartanian et al. (2013) and Kerameas et al. (2015) in that “appropriateness” indeed seems to be an important mediator of the influence of social norms on eating behavior. In the current research, both a descriptive norm (pilot experiment) and a norm with injunctive and descriptive characteristics (main experiment) were effective in reducing the portion size effect. An interesting avenue for future research will be to further compare the impact of descriptive and injunctive norm information on the portion size effect.

A limitation of this research is that we measured expected consumption and expected amount served, rather than actual consumption and actual amount served. We reasoned, however, that uncertainty about how much is appropriate to eat or serve will be quite similar in an actual consumption setting and a hypothetical one. As the main experiment showed, reducing the uncertainty about how big the portion size is by providing a picture did not diminish the portion size effect, and neither did it moderate the effect of the social norm manipulation. In addition, portion size preferences that were measured using food pictures (Wilkinson et al., 2012) or food replicas (Bucher et al., 2012), showed that these align well with actual consumption amounts. Also, various studies have shown that the portion size effect is also present when measuring expected consumption using consumption scenarios (Marchiori et al., 2014; Robinson et al., 2015; Versluis et al., 2015). We would further like to note that studying consumption quantity decisions in an online setting may also have some important advantages, for example that by answering the questions anonymously and in the comfort of their own home, participants may be more likely to imagine a real life eating situation and to answer honestly. Nonetheless, important determinants of consumption such as taste and feelings of satiety are not taken into account in scenario studies such as these, and hence conducting a similar study in which actual consumption is measured, is an important step for future research.

Another limitation is that we did not include control conditions without information about the normative relevance of the portion sizes. Hence, we cannot disentangle the specific effects of the in-group and majority versus the out-group and minority conditions compared to a standard portion size control condition. Although we expect that participants will typically assume that a given portion is appropriate and thus that a control condition without normative information would resemble the majority condition, including such a control condition will be an important direction for future research.

The current research suggests that the portion size acts as a social norm, since its effect was reduced when its normative relevance was reduced. However, this does not answer the question *why* the portion size acts as a social norm. We found evidence that the amount that was considered appropriate partially mediated the influence of portion size on the expected amount served. We do not know, however, whether this appropriateness refers to the perceived social effect of eating that amount, to the perceived healthiness, expected satiation, or to other ways in which participants might construe “appropriateness.” In addition to wanting to eat in line with what is socially acceptable (Herman et al., 2003), people might also use portion sizes as social information about correct amounts to eat in terms of nutrition (i.e., as informational social influence, see Deutsch and Gerard, 1955). An important avenue for further research will be to disentangle these meanings of appropriateness.

Furthermore, the amount that was considered appropriate only partially mediated the relation between portion size and expected amount served. It will thus also be important to determine exactly how important the wish to eat appropriately is when people determine how much to eat, and which other mechanisms lead them to use the portion size in their consumption decision. Such other mechanisms could for example be cognitive anchoring effects (Marchiori et al., 2014) and the learned tendency to finish the food on one’s plate (“plate cleaning”; Birch et al., 1987; Rolls et al., 2002; Wood and Neal, 2009).

Future research could also compare the effectiveness of different types of social information about the portion size as a means to reduce the portion size effect. In the current experiments, we used social groups (students, women) that were neutral with regard to eating behavior. Future studies could examine, for example, if using other social groups, such as fast-food lovers or those with a different body type than the participant, would make the normative relevance manipulation more effective. In addition, it would be interesting to further investigate whether a shared group membership manipulation (as we used in the pilot experiment) is more or less effective than a minority/majority manipulation (as we used in the main experiment). Finally, in the current research, we did not specify whether the portion size was considered too small or too large by the social group who did not find it appropriate. Clearly specifying that large portions are considered to be too large by relevant others could further improve the effectiveness of the normative relevance manipulation. In an experimental setting, however, this needs to be carefully weighed against the possibility of increasing demand effects.

## Conclusion

To our knowledge, these studies are the first to directly test whether the portion size signals to consumers how much is appropriate to eat and whether reducing this signaling value reduces the portion size effect. While we found evidence supporting the normative interpretation of the portion size effect, we should also note that reducing the normative relevance of the portion size did not fully remove the portion size effect in the main experiment. In addition, the effect size of the normative relevance manipulation was relatively small, and the amount that was considered appropriate only partially mediated the relation between portion size and expected amount served. Hence a critical evaluation of the various mechanisms that might together underlie the portion size effect remains important. Factors such as uncertainty, anchoring on the portion size (Marchiori et al., 2014), and the tendency to “clean the plate,” might all play a role in the effect, and future research might try to establish their importance in different situations and for different groups of consumers.

## Author Contributions

IV was involved in designing the study, collecting and analyzing and the data, and drafting the manuscript. EP was involved in designing the study, analyzing the data, and drafting the manuscript. Both authors approved the final manuscript.

## Conflict of Interest Statement

The authors declare that the research was conducted in the absence of any commercial or financial relationships that could be construed as a potential conflict of interest.

## Footnotes

1 The participant was presented with the percentage of women that approved of the portion sizes the participant was about to see. The participant was then asked to recall this percentage. If she filled in the wrong percentage, she was asked to re-read the information. If the participant then again filled in the wrong percentage, she could no longer continue, which happened to a total of 41 potential participants.
