# Supplementary material for: The Role of Social Norms in the Portion Size Effect: Reducing Normative Relevance Reduces the Effect of Portion Size on Consumption Decisions
Source: Front Psychol. 2016 May 31;7:756. doi: 10.3389/fpsyg.2016.00756 (PMC4885850; doi:10.3389/fpsyg.2016.00756)

Supplementary Material

The Role of Social Norms in the Portion Size Effect: Reducing normative relevance reduces the effect of portion size on consumption decisions

Iris Versluis*, Esther K. Papies

*** Correspondence:** Iris Versluis, info@irisversluis.nl

Figure S1. Main experiment portion size pictures: Pasta – small and large portion

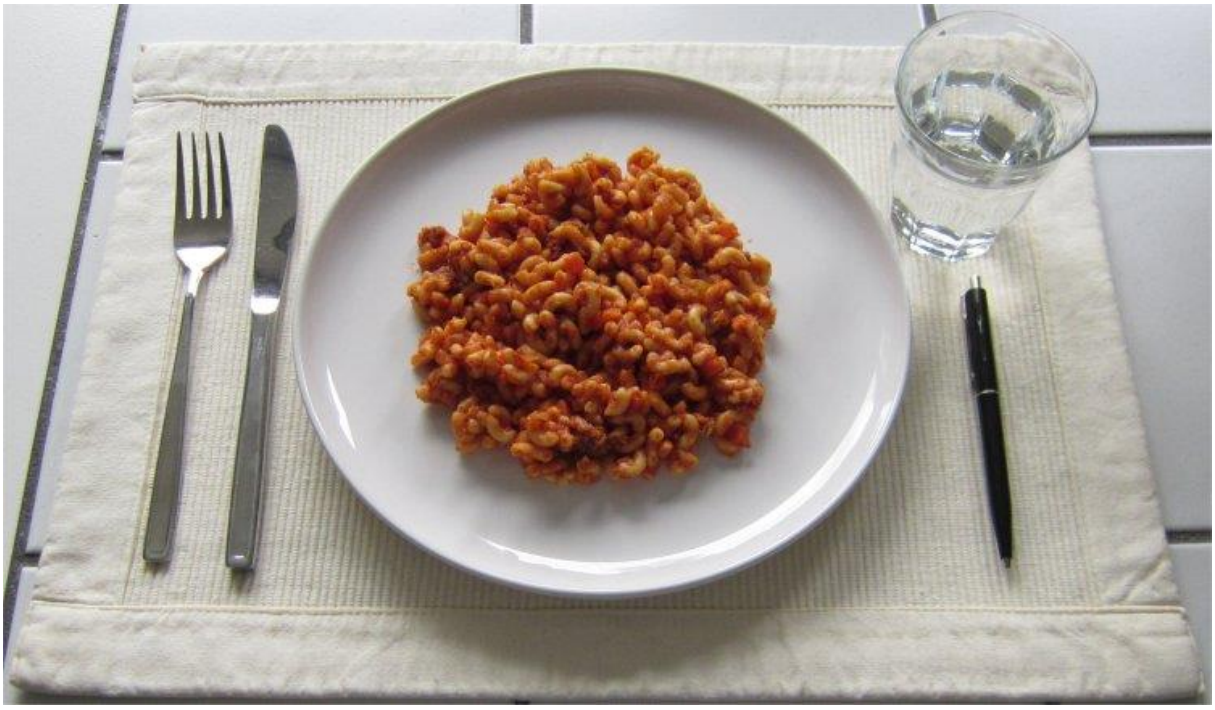

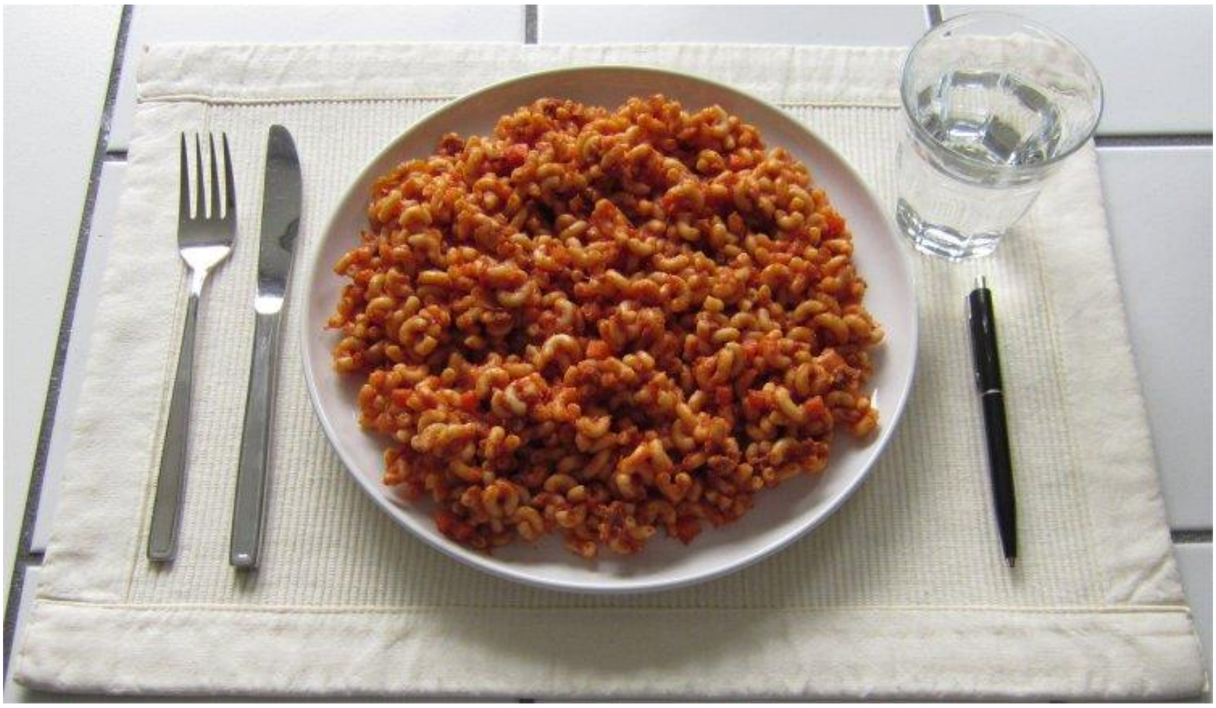

Figure S2. Main experiment portion size pictures: Indonesian fried rice - small and large portion

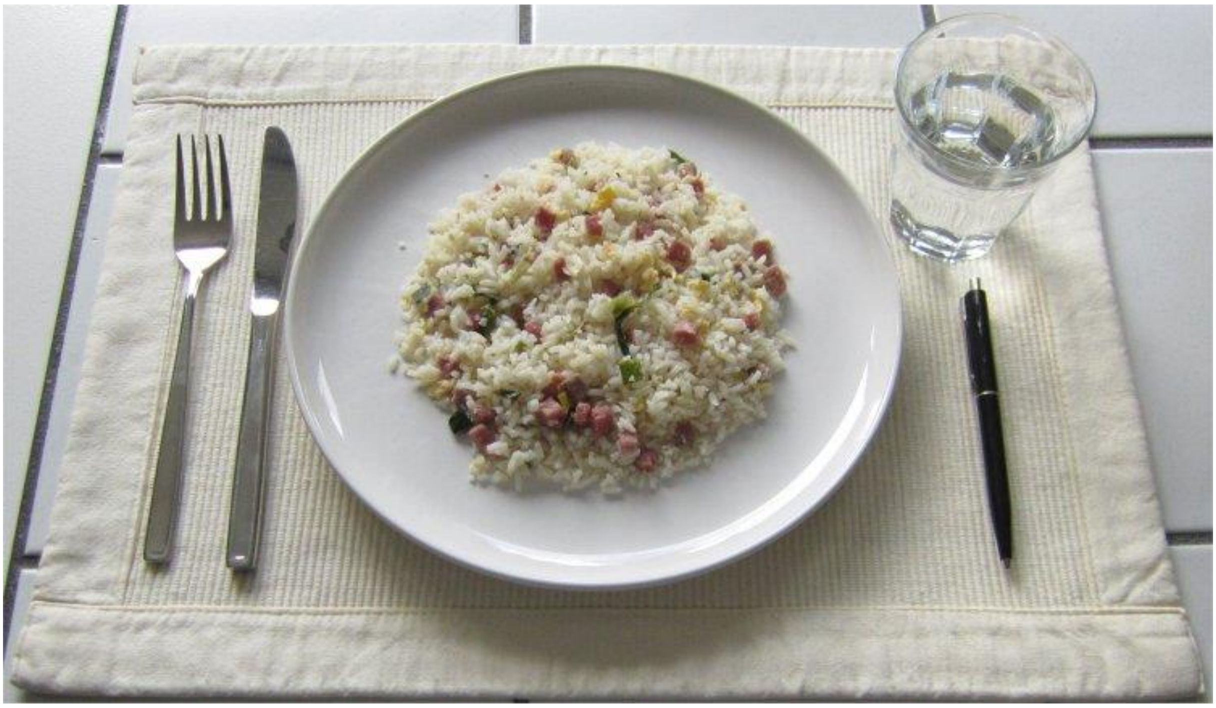

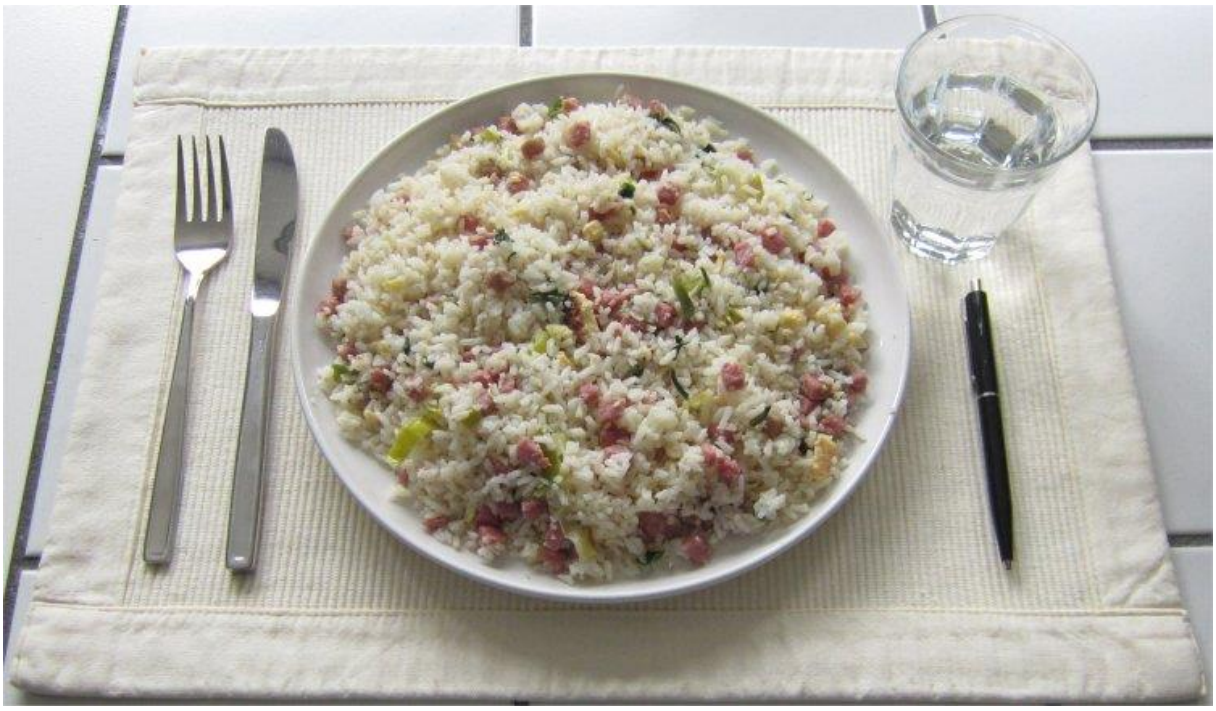

Figure S3. Main experiment portion size pictures: Mini ginger cookies – small and large portion

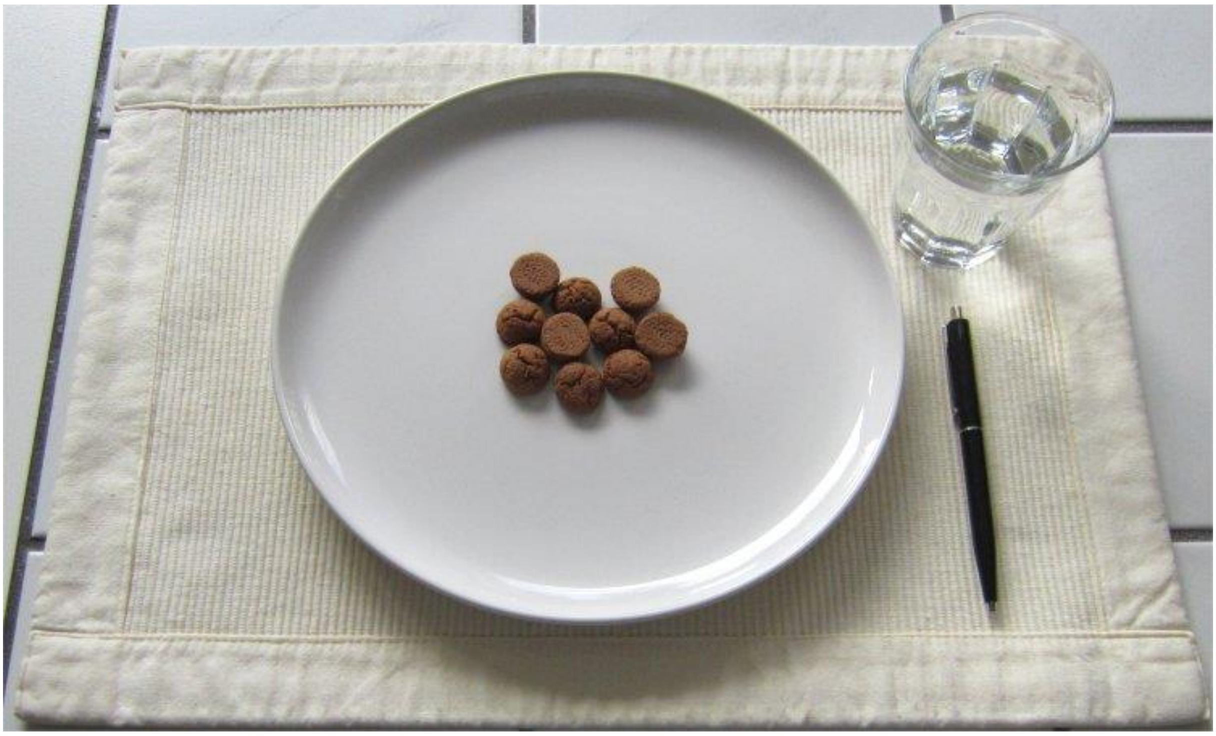

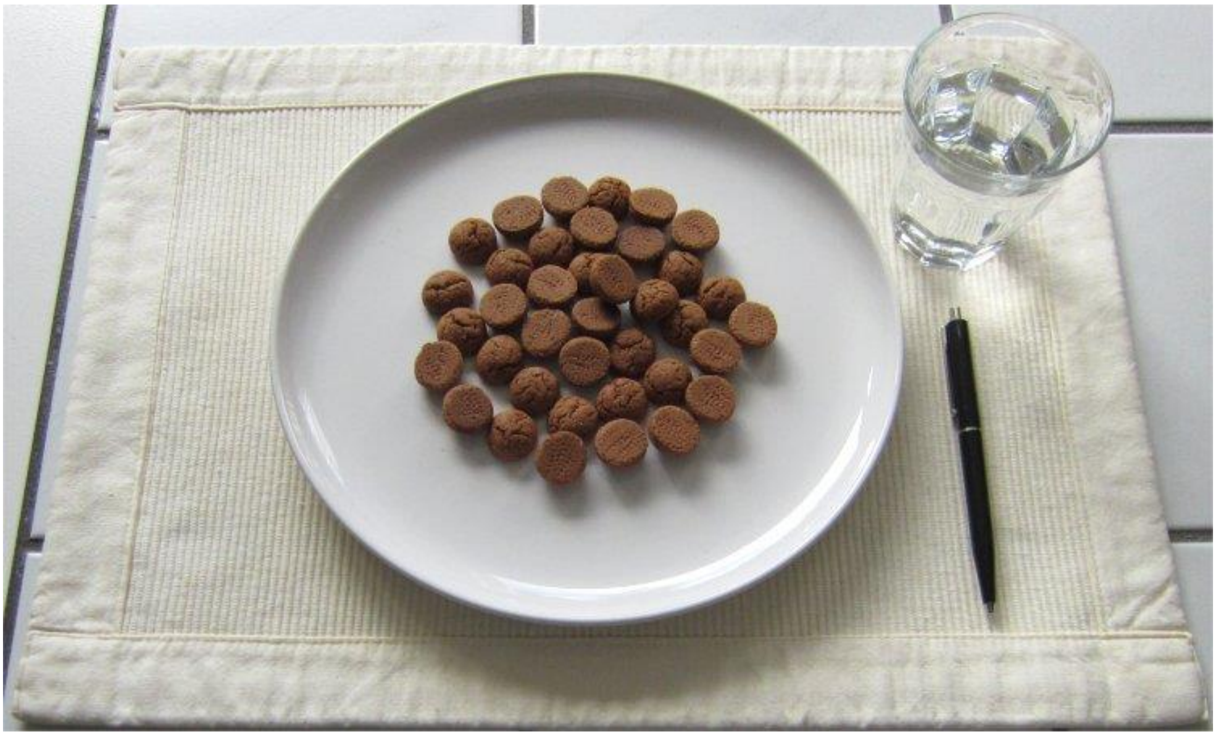

Figure S4. Main experiment portion size pictures: Chips– small and large portion

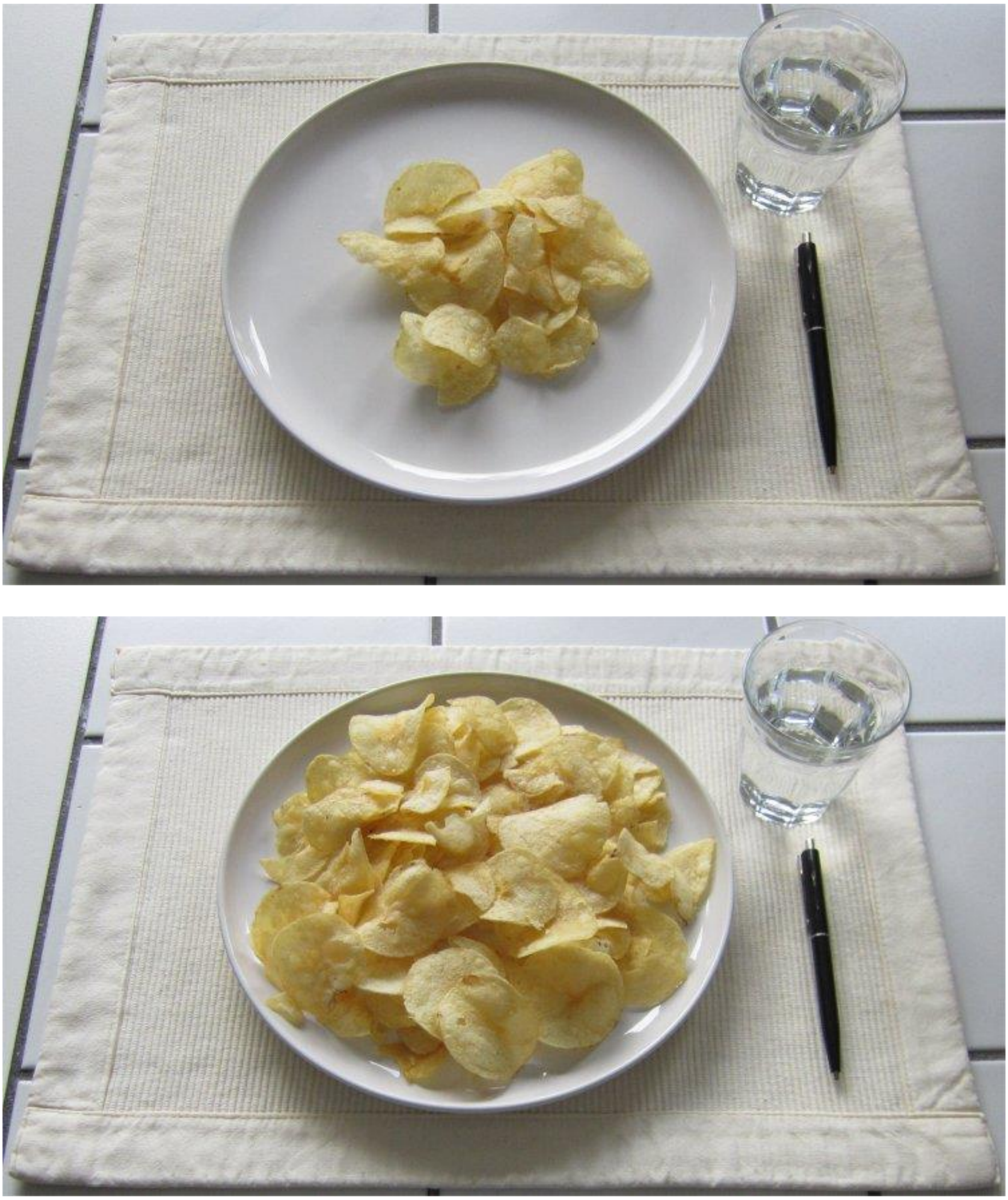

Supplement: Supplementary file 1 [file Presentation_1.PDF]
