# Supplementary material for: The Role of Social Norms in the Portion Size Effect: Reducing Normative Relevance Reduces the Effect of Portion Size on Consumption Decisions
Source: Front Psychol. 2016 May 31;7:756. doi: 10.3389/fpsyg.2016.00756 (PMC4885850; doi:10.3389/fpsyg.2016.00756)
Supplement: Supplementary file 2 [file Table_1.DOCX]

Supplementary Material

**The Role of Social Norms in the Portion Size Effect:**

**Reducing normative relevance reduces the effect of portion size on consumption decisions**

Iris Versluis*, Esther K. Papies

*** Correspondence:** Iris Versluis, [info@irisversluis.nl](mailto:info@irisversluis.nl)

Table S1: Additional measures included in main experiment

| **Measure** | **Scale** |
| --- | --- |
| Statements regarding the extent to which participants make their own portion size choices / are influenced by others:   - I know very well what a suitable portion size for me is - I often check the package to see what the right portion size is - I honestly do not know if the portion sizes that I eat are suitable for me - To determine how much I can eat of something, I look at what others eat - I don’t care how much others eat, I determine how much I eat | 1 = *strongly disagree* to 7 = *strongly agree* |
| Identification with eating habits of Dutch women:   - My eating habits are very similar to Dutch women - I have different eating habits than most Dutch women - My eating habits are healthier than those of the average Dutch woman - Most Dutch women are knowledgeable about eating healthily - Most Dutch women are knowledgeable about eating tasty | 1 = *strongly disagree* to 7 = *strongly agree* |
| What is your opinion of a portion of <insert amount> grams of <insert food>? | 1 = *way too little* to 7 = *way too much* |
| Did you expect that the percentage of women that found the portion appropriate would have been higher or lower? | 1 = *much lower*  to 5 = *much higher* |
| How often do you prepare an evening meal with fresh ingredients? | *0 – 7* times a week |
| How often do you use a kitchen scale when preparing a meal?  How often do you use a measuring cup when preparing a meal? | 1 = *never* to 7 = *always* |
| Currently pregnant or breastfeeding? | Yes/no |
| Currently using medicines that influence appetite? | Yes/no |
| Do you follow any of the following diets? | Participants that followed diets displayed in italics were not allowed to continue with the questionnaire.  *cow-milk free* / *lactose free* / *diet for allergy nuts and peanuts* / *diet for diabetics* / protein restricted / fat or cholesterol restricted / *colour agent free* / energy or protein rich diet / I follow none of the above specified diets |
| Now or in the past diagnosed with an eating disorder? | Yes/no/no answer |
| Highest completed education | Lager onderwijs (LO) / Lager beroepsonderwijs (LBO) of Voorbereidend Middelbaar Beroepsonderwijs (VMBO) / MAVO / HAVO or VWO / MBO / HBO / Universitair / Other |
| Living situation | Living alone / Living with parents or family / Living with friends or students / Married or living with partner |
